# Exploring various models of health coaching for improving blood pressure control among patients with hypertension attending primary health care settings: A scoping review

**DOI:** 10.51866/rv.789

**Published:** 2025-02-27

**Authors:** Arief Alamsyah, Fatwa Sari Tetra Dewi, Vita Yanti Anggraeni, Iqbal Sholahudin Maududdy

**Affiliations:** 1 MD, MHA, Doctoral Program in Medicine and Health Science, Faculty of Medicine, Public Health and Nursing, Universitas Gadjah Mada, Farmako Sekip Utara St, Yogyakarta, Indonesia.; 2 Department of Family Medicine, Faculty of Medicine, Universitas Brawijaya, Veteran St, Malang, Indonesia. Email: alamsyah.fk@ub.ac.id; 3 MD, MPH, Ph.D, Department of Health Behavior, Environment and Social Medicine, Faculty of Medicine, Public Health and Nursing, Universitas Gadjah Mada, Farmako Sekip Utara St, Yogyakarta, Indonesia.; 4 MD, Ph.D, Department of Internal Medicine, Faculty of Medicine, Public Health and Nursing, Universitas Gadjah Mada, Farmako Sekip Utara St Yogyakarta, Indonesia.; 5 MD, Faculty of Medicine, Universitas Brawijaya, Veteran St, Malang, Indonesia.

**Keywords:** Blood Pressure, Coaching, Health, Patient, Primary Health Care

## Abstract

**Introduction:**

This study aimed to explore various models of health coaching used for blood pressure control in patients visiting primary care settings, along with the underlying theories. Additionally, the study sought to identify individuals serving as health coaches and the effect of health coaching on blood pressure control and risk factors.

**Methods:**

This scoping review followed the PRISMA-ScR guidelines and utilised several databases including PubMed, ScienceDirect, ProQuest, Scopus and Web of Science. The search focused on articles published from January 2012 to July 2024. Eligible articles were examined to identify the forms of health coaching, the backgrounds and roles of health coaches and the outcomes of health coaching.

**Results:**

An initial search yielded 963 articles, of which 16 were selected for the review. Most studies (n=10) showed that health coaching was used in conjunction with other strategies (multicomponent). The most frequently applied approach was phone coaching (n=9). The majority of the interventions were completed within 6 months (n=4). Eight studies indicated that most health coaches came from health backgrounds including nurses, pharmacists and family doctors. Positive clinical outcomes, such as decreased systolic and diastolic blood pressures or systolic or diastolic blood pressure alone, were documented in all included investigations. The non-clinical outcomes varied.

**Conclusion:**

Health coaching is a promising approach for controlling blood pressure in primary care settings. This study highlights the importance of designing the form, time and staff for conducting effective health coaching in primary care settings.

## Introduction

Non-communicable diseases (NCDs) such as cardiovascular disease, diabetes mellitus and cancer have become a global concern as they accounted for 200 million deaths among people aged 30 - 70 years in the last two decades. These diseases most frequently occur in low- and middle-income countries (LMICs)^1^. Hypertension is a significant risk factor for cardiovascular disease. In Indonesia, a multinational survey showed that the prevalence of hypertension remained high, reaching 34.5%. This indicates the need for more effective control of hypertension to reduce mortality and morbidity due to cardiovascular disease.^2,3^

Various methods have been developed to improve hypertension awareness and control. An education-based strategy for lifestyle modification and medication adherence is still considered more cost-effective than other approaches.^4^ Primary care services, as the first line of health services, are the spearhead in the early detection, risk management and treatment of NCDs, including hypertension^5,6^ For example, in Indonesia, education and promotion of healthy living in primary care services are part of the activities of the integrated health posts for NCDs *(PTM Posbindu)* and integrated health posts for older people *(Posyandu Lamia),* along with anthropometric examinations, blood pressure measurements and blood sugar and cholesterol examinations. This approach can increase the awareness of patients with hypertension and help them understand their condition. However, a study found that awareness and participation rates in this activity did not correlate significantly with blood pressure control, where only 25% of participants could achieve a blood pressure of 140/90 mmHg^7,8^. A more intensive approach is needed to manage blood pressure and lifestyle among patients with hypertension.

Health coaching is an approach to health education and promotion with coaching principles to improve patient well-being and enable patients to achieve their targets in controlling their condition.^9^ The task of a coach is to assist patients in setting goals, creating an agenda for change, monitoring and evaluating progress and identifying obstacles that may arise.^5^ Currently, there are many variations of health coaching for chronic conditions in terms of the modality (e.g. face-to-face, telephone or text message) and duration of health coaching, the background of training education for coaches and the effectiveness in improving physiological, behavioural and social outcomes.^10^

This scoping review aimed to explore the forms of health coaching conducted for improving blood pressure control among patients visiting primary care settings, along with the underlying theories. The limitations or challenges of each form were also reviewed. Furthermore, this review sought to identify health coaches and the impact of health coaching on blood pressure control and risk factors.

## Methods

This scoping review followed the PRISMA- ScR guidelines to review studies relevant to the research objectives. The steps were based on the five-step guidelines from Arksey and O’Malley: 1) identification of research questions, 2) identification of relevant studies, 3) selection of articles, 4) synthesis of data and 5) reporting of data and drawing of conclusions.

### Data sources

The population/participants, concept and context (PCC) approach from the Joanna Briggs Institute Manual was used to find relevant studies. The population was patients with hypertension; concept, health coaching; and context, primary healthcare.

The search focused on the last 10 years to obtain the most recent studies. Appropriate literature published from January 2012 to July 2024 was searched using several databases including PubMed, ScienceDirect, ProQuest, Scopus and Web of Science. The following keywords were used: (‘hypertension’ OR ‘high blood pressure’ OR ‘elevated blood pressure’) AND (‘health coaching’ OR ‘wellness coaching’) AND (‘primary health care’ OR ‘primary care’ OR ‘community health center’ OR ‘general practitioner’ OR ‘family physician’). These keywords were combined in each database to achieve the maximum study results in terms of the number of studies found.

### Inclusion and exclusion criteria

A literature review of studies that tested the effectiveness of health coaching as an intervention for hypertension control in primary care settings was conducted. The inclusion criteria were as follows: 1) English and Indonesian literature; 2) any age range of participants; 3) participants specialising only in patients with hypertension without other NCDs such as obesity or diabetes mellitus; 4) health coaching outcomes, including blood pressure changes, lifestyle changes or self-efficacy; and 5) various methods of health coaching, including face-to-face or telephone coaching.

Conversely, the exclusion criteria were as follows: 1) grey literature and unpublished research since unpublished articles are not peer- reviewed, which can affect the quality of the findings; 2) research conducted outside primary care settings including clinics, primary care centres and community health centres where general practitioners and family physicians work; and 3) protocols and other study designs.

The researcher was assisted by a reviewer (IQ) to assess the identified articles independently. The title and abstract of the articles were screened. After the initial screening, the full text of the identified articles was retrieved to review their content and assess which of them were eligible for inclusion. When there was any discrepancy in the assessment of the articles, a discussion was conducted to make the final decision.

### Data extraction

The researcher (AA) filtered the titles and articles of the identified articles. AA screened the content of some articles both alone and together with an independent reviewer (IQ). When there was any difference in the determination of the eligible articles, it was resolved through a discussion to reach an agreement.

The following data were extracted from the selected articles: 1) name of the researcher and country where the study was conducted; 2) participants and setting of the study; 3) design of the study; 4) theoretical basis of health coaching; 5) details of health coaching including the modality/form, duration and implementation (health coach); and 6) outcome of health coaching. The data extracted were presented in a tabular format.

## Results

### Search results

**Figure 1.**
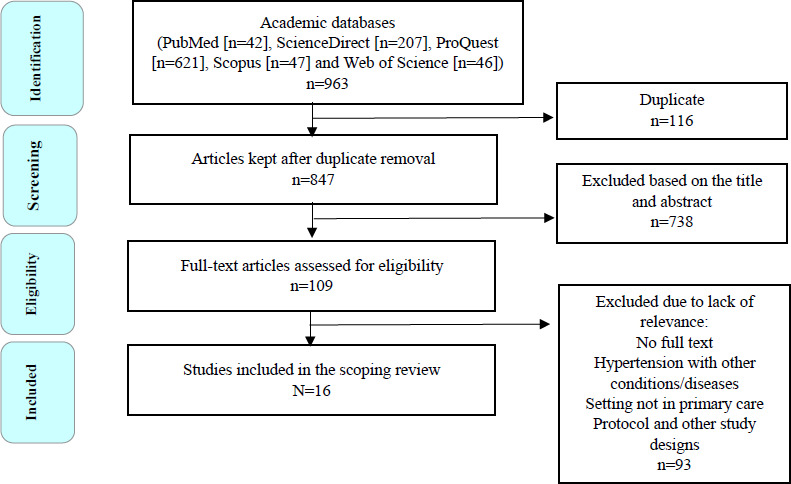
PRISMA-ScR flow diagram of article identification, screening, eligibility and inclusion. *Studyperiod and location*

The initial search using the five databases yielded 963 articles. After a duplicate check, 847 articles were left. Title and abstract screening yielded 109 articles, of which their full text was retrieved and evaluated by two reviewers. After reviews and discussions, 16 articles were finally included according to the inclusion and exclusion criteria (Figure 1).

### Study period and location

Most studies (n=10, 62.5%) were conducted in the United States.^11-20^ Two studies were conducted in China^21,22^ and the remaining studies in Argentina,^23^ India,^24^ Australia^25^ and Egypt.^26^ The most common study period was 2017 (n=5, 31.25%).^12-15,22^ The earliest study was conducted in 2012 in the United States,^11^ while the most recent study was conducted in 2024 in Egypt^26^ (Table 1).

**Table 1:** Health coaching interventions.

Source and study setting	Study design	Sample size	Theory	Duration	Health coaching intervention
Margolius et al. (2012) United States	Randomised between groups; pretest-posttest design	N=237, including a group with home titration (n=129) and a group without home titration (n=108)	No information	6 months	Form of intervention: - Weekly phone calls to discuss overall well-being, adherence to action plans and blood pressure targets - Health coaching combined with home blood pressure monitoring and home titration of antihypertensive drugs Health coach: - 10 University of California, San Fransisco (UCSF) employees and volunteers with non-medical bachelor’s degrees - Health coaches received 16 to 20 h of training in hypertension, its medications and lifestyle behaviour changes, including medication adherence counselling.
Milani et al. (2017) United States	Pretest-posttest design	N=556, including an intervention group (n=156) and a control group (n=400)	No information	90 days	Form of intervention: - Coaching was conducted over the phone, discussing therapy plans and motivating patients to actively use the website on a digital platform in which educational materials about hypertension and lifestyle changes through videos and handouts were provided. Health coach: - Pharmacist
Halladay et al. (2017) United States	Non-randomised prospective cohort trial	N=525	Literacy- sensitive process combined with coaching	24 months	Form of intervention: - The intervention was conducted by combining health literacy improvement and phone coaching as well as a media handout. - Health literacy improvement was facilitated through videos, role plays and literature reviews. - The topic of phone coaching was medication compliance, physical activity, healthy diet, blood pressure measurement, weight, social barriers, stress control, cigarettes and alcohol. - Each participant was coached once a month for 15–20 min. Health coach: - Trained health coach
He et al. (2017) Argentina	Cluster randomised trial	N=1432, including an intervention group (n=743) and a control group (n=689)	No information	18 months	Form of intervention: - An 18-month multicomponent intervention programme was conducted in intervention clinics, involving several main components, such as home interventions, coaching by doctors, weekly texts and family-based interventions (home visits). Home interventions: - Community health workers were trained to provide health training, home blood pressure monitoring and audits. They visited participants’ homes monthly for the first 6 months and once every 2 months thereafter. Coaching by physicians and blood pressure feedback: - Physicians were trained in hypertension management and were given feedback based on blood pressure monitoring data at home by community health workers. Coaching via weekly text messages: - Participants received weekly text messages to support lifestyle changes and medication adherence. Family-based interventions: - These interventions started with an initial home visit that involved all family members to discuss the prevention and treatment of hypertension. Subsequent visits provided tailored coaching on lifestyle modifications and medication adherence. Health coach: - Community health worker and doctor
Yan-Jin et al. (2017) China	Randomised controlled trial	N=102, including an intervention group (n=53) and a control group (n=49)	No information	6 months	Form of intervention: - Combination of community-based group education (3 months) with coaching sessions (3 months) on systolic blood pressure and heart disease risk factors. Health coach: - Two community nurses
Crittenden et al. (2017) United States	Prospective pre-test–post-test design	N=21	No information	8 weeks	Form of intervention: - Phone coaching every 2 weeks to analyse the progress and perceived obstacles patients feel in modifying their hypertension management lifestyle Health coach: - Nurse
Ursua et al. (2018) United States	Randomised controlled trial	N=240, including an intervention group (n=112) and a control group (n=128)	Health belief model and social support theory	8 months	Form of intervention: - Combination of monthly interactive health education methods (group/individual) for 90 min - Follow-up in the form of one-on-one coaching with home visits - Coaching was a goal-setting plan, helping patients access primary care services, receive referrals needed for mental health services and quit smoking. - Sometimes, coaching was also conducted over the phone. Health coach: - Community health worker
Wu et al. (2018) United States	Secondary data analysis	N=525	No information	12 months	Form of intervention: - Phone coaching was conducted every month per patient to review the progress of blood pressure medication compliance through self-determined goal-setting. Health coach: - Officers who received training using scripts and role plays
Milani et al. (2020) United States	Pretest–posttest design	N=803	No information	12 months	Form of intervention: - Combination of digital tools and health coaching. Patients took blood pressure measurements sent via Bluetooth or smartphone. Patients received a text message when they did not send data for 8 days. - Coaching was conducted via phone, discussing lifestyle and treatment and motivating patients to be active on the website to watch videos and read handouts. Patients received monthly reports on their progress in lifestyle changes. Health coach: - Pharmacist and health coach
Poggio et al. (2019) Argentina	Cluster randomised trial	N=1954	Motivational interviewing and stages of change model	18 months	Form of intervention: - Coaching was based on a family approach through home visits every 1–2 months. Each coaching was conducted for 60 min. - Text messages were sent to patients and family members weekly through a web-based platform. A text message is a one-way outgoing message system. Health coach: - Community health workers trained in motivational interviewing and the stages of change model for 2 days
Odemelam et al. (2020) United States	One-group pretest–post test design	N=29	Social cognitive theory	8 weeks	Form of intervention: - Phone coaching to each patient for 15 min, once per week; phone coaching on the importance of medication adherence, drug side effects, low-salt diet/dietary approaches to stop hypertension (DASH) diet, weight management, physical activity, alcohol limitation and cigarette reduction - Patients were also given tools for self-blood pressure monitoring at home. Health coach: - No information
Singh et al. (2021) Australia	Controlled group studies	N=30, including an intervention group (n=20) and a control group (n=10)	Transtheoretical model or stages of change model	3 months	Form of intervention: - Each patient received one face-to-face coaching per month, covering three risk factors, namely diet, physical activity and medication management. Health coach: - Pharmacist
Kannure et al. (2021) India	Quasi-experiment between groups	N=13,184	No information	12 months	Form of intervention: - The intervention was divided into two parts: intensive intervention (monthly phone calls and free medicine) and light intervention (quarterly phone calls and free medicine). - Telephone conversations about medication adherence, lifestyle education, uncontrolled blood pressure complications and encouragement to visit the practitioner for regular blood pressure measurements Health coach: - Trained nurse
Sun et al. (2022) China	Cluster randomised trial	N=33,995, including an intervention group (n=15,414) and a control group (n=14,500)		18 months	Form of intervention: - Health coaching was conducted individually or in groups every month. During the COVID lockdown, coaching was performed via telephone. Health coaching was combined with self-monitoring of blood pressure at home (two to three times a week) and incentives to buy drugs for low-income patients. Health coach: - Village doctor who was trained for 3 days in the importance of hypertension control, standard methods of measuring blood pressure, stepwise protocol on hypertension management and health coaching
Islam et al. (2023) United States	Randomised controlled trial	N=303, including an intervention group (n=159) and a control group (n=144)	No information	6 months	Form of intervention: - Combination of group education conducted four times with individual coaching via phone every 2 weeks Health coach: - Community health worker
Abbas et al. (2024) Egypt	Quasi- experimental pretest-posttest control group design	N=141, including an intervention group (n=70) and a control group (n=71)	No information	6 months	Form of intervention: - Health coaching was conducted face-to-face and via phone. Face-to-face coaching was conducted every 2 months for 20-30 min. Phone coaching was performed twice a week (biweekly) for 15-30 min. Health coach: - Family doctor

### Study design and participants

The study design of the selected articles varied. The majority were randomised trials (n=3),^14,21,23^ randomised controlled trials (n=3, 18.75%)^16,20,22^ and pre-test-post-test studies (n=3).^12,15,18^ Two studies used a randomised design with pre-tests and post-tests between groups. One study each was a quasi-experiment between groups^24^, a non-randomised prospective cohort trial,^13^ a control group study^25^ and a secondary data analysis^17^ (Table 1).

Participants all came from primary care settings with varying proportions. The smallest number of participants was 21 in the United States,^15^ while the largest number was 13,184 in India.^24^

### Interventions

Forms of the interventions The review revealed that health coaching for improving blood pressure control in primary care settings was conducted either as a single intervention (n=6, 37.5%)^11,15,19,24-26^ or in combination with other interventions (multicomponent) (n=10, 62.5%).^11-14,16-18,20-22^ Another intervention included the use of a digital platform (website) (n=2, 12,5%)^12,23^ and digital tools.^18^ Multicomponent interventions included family education and family-based interventions,^14^ community-based education,^22^ health literacy improvement (through videos and role plays),^13^ self-monitoring of blood pressure at home and incentives to buy drugs for patients with low income.^21^

The forms of health coaching conducted for managing hypertension in primary care settings were face-to-face coaching, telephone (phone) coaching and text messaging. Most coaching was conducted via phone (n=9, 56.25%).^11-13,15,17-19, 21,24^ Phone coaching and face-to-face coaching were combined in three studies (n=3, 18.75%).^20,22,26^ A smaller proportion involved only face-to-face coaching (n=2, 12.5%),^16,25^ while face-to-face coaching combined with weekly text messaging was provided in two studies (12.5%)^14,23^ (Table 2).

**Table 2:** Characteristics of the health coaching interventions conducted in primary care settings (N=16).

Characteristics	n (%)
Country of the study	
Developed countries	12 (75)
Low- and middle-income countries	4 (25)
Components of the intervention	
Combined health coaching (multicomponent)	10 (62.5)
Health coaching only	6 (37.5)
Forms of health coaching	
Phone coaching and face-to-face coaching	3 (18.75)
Phone coaching	9 (56.25)
Face-to-face coaching	2 (12.5)
Face-to-face coaching and text messaging	2 (12.5)
Duration	
0–6 months	8 (50)
7–12 months	4 (25)
13–18 months	4 (25)
Coach	
Doctor	2 (12.5)
Nurse	3 (18.75)
Pharmacist	3 (18.75)
Community health worker	3 (18.75)
Doctor and community health worker	1 (6.25)
Other trained personnel	3 (18.75)
No information	1 (6.25)
Location	
Patient’s home	4 (25)
Clinic, community health centre and general practitioner/family physician	7 (43.75)
Community pharmacist	1 (6.25)
No special place/no information	4 (25)
Underlying theory	
Literacy-sensitive process	1 (6.25)
Health belief model and social support theory	1 (6.25)
Stages of change model and motivational interviewing	1 (6.25)
Social cognitive theory	1 (6.25)
Stages of change model	1 (6.25)
No information	11 (68.75)

**Duration and frequency of the interventions**

The duration of the interventions varied from 8 weeks (2 months) to 18 months. Most interventions were performed within 6 months (n=4),^11^,^20^,^22^,^26^ followed by 18 months (n=3, 18.75%),^14,21 23^ 12 months (n=3, 18.75%),^17,18^’^24^ 3 months (n=2, 12.5%)^14,25^ and 2 months (n=2, 12.5%)^15,19^ (Table 2).

The frequency of phone coaching varied: once a week in two studies,^11,19^ monthly in one study,^17^ twice a week (biweekly) in one study^26^ and once every two weeks in one study.^15^ Most studies did not mention the frequency.

**Theoretical basis of the interventions**

Not all articles reviewed mentioned the theoretical basis used in their coaching interventions. Some theories mentioned included the health belief model, the social support theory, motivational interviewing and the transtheoretical model (stages of change model). Most studies (n=2, 12.5%) used the transtheoretical model (stages of change model), one of which combined it with the motivational interviewing approach^23,25^ (Table 1).

**Health coaches**

Both health and non-health workers conducted health coaching, although not all articles mentioned the background of health coaches. Most coaches came from health backgrounds, especially nurses (n=3, 18.75%),^15,22,24^ pharmacists (n=3, 18.75%)^12,18,25^ and family and village doctors (n=2, 12.5%)^21,26^ (Table 2). Health coaches from non-health backgrounds included community health workers (n=3, 18.75%)^14,16,20^ and professionals from other fields. On average, they received training for 2 days using materials about hypertension control and risk factors and how to conduct coaching (Table 1).

**Outcomes of the interventions**

Coaching was conducted to help coachees reach their blood pressure goal established during goal-setting. Accordingly, coaching focused on managing lifestyle and medication compliance. The results of health coaching varied. Most of the articles stated a decrease in systolic and diastolic blood pressures (n=8, 50%), followed by a decrease in systolic (n=6, 37.5%) or diastolic blood pressure alone (n=1, 6.25%) (Table 3). Lifestyle influences also differed. One article mentioned an increase in the habits of eating fruits and vegetables and doing physical activities. However, the interventions did not affect alcohol intake, smoking or weight gain.^23^ Additionally, the coaching agenda covered a number of topics, including identifying social barriers,^13^ assisting patients in accessing primary care physician services and mental health support^16^ and teaching them how to selfmonitor their blood pressure and self-titrate their medications (home titration).^11^

**Table 3:** Overview of the forms and outcomes of the interventions.

Study	Form of health coaching	Duration of health coaching	Systolic blood pressure	Diastolic blood pressure	Other outcomes
Margolius et al., 2012	Telephone coaching	Weekly	+		Decrease in clinic visits
Milani et al., 2017	Telephone coaching	Not reported	+	+	Decrease in salt consumption
Halladay et al., 2017	Telephone coaching	Once per month for 15–20 min			
He et al., 2017	Face-to-face coaching and text messaging	Monthly face- to-face coaching and weekly text messaging	+	+	
Yan-Jin et al., 2017	Telephone coaching and face- to-face coaching	One face-to-face coaching and two phone calls every month	+		Decrease in cholesterol levels, BMI and depression as well as increase in HRQoL
Crittenden et al., 2017	Telephone coaching	Once every 2 weeks	+		
Ursua et al., 2018	Face-to-face coaching	Once a week	+	+	Appointment keeping
Wu et al., 2018	Telephone coaching	Once a week		+	Medication compliance
Milani et al., 2020	Telephone coaching	Not reported	+		
Poggio et al., 2019	Face-to-face coaching and text messaging	The initial 90- min face-to-face coaching was continued one to two times monthly for 60 min per session. Weekly text messaging			Increase in the habits of eating fruits and vegetables and doing physical activities
Odemelam et al., 2020	Telephone coaching	Once a week for 15 min	+	+	Increase in self-efficacy, consumption of a low-salt diet, physical activity and cigarette smoking
Singh et al., 2021	Face-to-face coaching	Monthly	+		Medication compliance
Kannure et al., 2021	Telephone coaching	Monthly and quarterly	+	+	
Sun et al., 2022	Telephone coaching		+	+	Medication compliance
Islam et al., 2023	Face-to-face coaching and phone coaching Face-to-face coaching and phone coaching	Once every 2 weeks	+	+	All self-management activities excluding BMI
Abbas et al., 2024	Face-to-face coaching and phone coaching Face-to-face coaching and phone coaching	Face-to-face coaching every 2 months for 20–30 min Phone coaching twice a week for 15–30 min	+	+	Medication, low-salt diet and weight management

+ Significant change after the interventions.

## Discussion

This scoping review explored the forms of health coaching, the individuals serving as health coaches and the effects of health coaching on blood pressure and risk factors. Herein, most articles indicated telephone coaching as the most frequently used modality. This result is different from previous findings showing face-to-face coaching as the dominant modality.^27^ However, owing to its effectiveness in controlling blood pressure, telephone coaching can be considered, especially in conditions of limited resources, which do not allow for face-to-face coaching. Coaching only by phone without face-to-face communication can also positively influence patients in cases of hypertension and other cases. According to Lawson et al., telephone coaching (without face-to-face communication) can positively affect stress levels, diet, physical activity and emotional health, including readiness to change in many cases of chronic diseases, including depression, congestive heart failure, diabetes, hyperlipidaemia, hypertension, osteoporosis, asthma and low back pain.^28^ Meng et al. also pointed out that phone coaching was the most effective approach.^29^ In addition, health coaching is often combined with digital technology, such as mobile applications, physical activity-measuring devices and m-health, to manage patient problems and risk factors.^30-35^ This review also aligns with a scoping review of telemedicine interventions for hypertension in LMICs, where telephones are the most frequently used technology.^36^ This shows that telephone interventions are no longer an obstacle in LMICs.

Based on the results of this review, health and non-health workers can function as health coaches for hypertension control in primary care settings. The involvement of non-health workers in primary care services is important because of many limitations, including the limitations of human resources, especially healthcare workers, who have multiple roles and responsibilities. If non-health workers can carry out health coaching, it will be more effective and efficient.^8^ Some of the articles in this review showed that the involvement of non-health workers, such as community health workers, was quite effective. However, this must be studied further because other studies show different results. Shah et al. found contrasting results, where in the Enhanced Primary Care Programme, the involvement of non-health workers and health coaches was not substantially effective because of insignificant outcomes on several parameters, even though they had received training for 2 days.^37^ Another aspect that was not explicitly explained in all articles was the comparison between the number of health coaches and patients as coachees, so it was not possible to draw an ideal comparison between them. This comparison is important if health coaching is applied, especially in LMICs with various limitations.

All included studies in this review reported positive clinical outcomes including decreased systolic and diastolic blood pressures and systolic or diastolic blood pressure alone. The non-clinical outcomes varied. Similarly, the systematic and methodological analysis conducted by Meng et al. showed that health coaching significantly reduced systolic and diastolic blood pressures. As for lifestyle and risk factors, only dietary habits and self-efficacy were significantly affected.^29^

Finally, a detailed exploration of health coaching models showed that telephone coaching was the most frequently used model, followed by face-to-face coaching and text messaging. However, this study cannot fully provide a detailed overview of the method and duration of coaching because not all studies reported them. One promising aspect of health coaching for hypertension in the future, especially in primary care settings, is that health coaches do not have to come from health backgrounds. Health coaching can also be conducted by other workers, even community health workers who receive standardised training. This can be a solution to the limited resources in primary care services and a material for future studies to compare the outcomes between coaching conducted by health and non-health workers.

This scoping review has several limitations, including the fact that studies not published in English and Indonesian were excluded. The studies also varied greatly regarding their study location, study design, sample demographics, interventions and outcomes. This makes it difficult to generalise the results to the country where the study was conducted and the specific population. The intervention outcomes greatly differed, especially the non-clinical outcomes other than blood pressure (systolic and diastolic). However, this study suggests that health coaching for hypertension is more of a component within a multicomponent approach rather than a standalone health coaching method. Consequently, the outcomes obtained cannot be solely attributed to health coaching because several other components are involved.

## Conclusion

Health coaching is a promising approach for controlling blood pressure in primary care settings. This review emphasises the importance of designing the form, time and staff for conducting effective health coaching in primary care settings.
